# Systemic Endothelial Function in Primary Open-Angle Glaucoma

**DOI:** 10.1155/2014/529082

**Published:** 2014-06-18

**Authors:** Mustafa Atas, Hasan Basri Arifoglu, Arzu Seyhan Karatepe Hashas, Bahadır Sarli, Suleyman Demircan, Ayse Ozkose, Altan Goktas

**Affiliations:** ^1^Department of Ophthalmology and Vision Science, Kayseri Training and Research Hospital, Sanayi Mah. Atatürk Boulevard Hastane Street, No. 78, 38010 Kayseri, Turkey; ^2^Department of Cardiology, Kayseri Training and Research Hospital, 38010 Kayseri, Turkey

## Abstract

*Objective*. We aimed to assess peripheral vascular endothelial function in open-angle glaucoma (POAG) by measuring flow-mediated dilatation (FMD). *Materials and Methods*. The study included 20 cases with POAG (group 1, mean age 58.68 ± 13.3 years) and 30 healthy individuals (group 2, mean age 58.68 ± 13.6 years). In all cases, responses of endothelial function were assessed by a cardiologist through measurement of FMD following brachial artery occlusion. *Results*. Mean percent of FMD, an indicator of endothelial function, was found to be 11.9 ± 4.2% in group 1 and 12.3 ± 4.4% in group 2 (*P* = 0.86). *Conclusion*. No impairment in systemic vascular function of cases with POAG suggests that POAG could be a local disorder rather than being a component of systemic disease.

## 1. Introduction

Glaucoma is an irreversible optic neuropathy characterized by specific optic disc changes and corresponding visual field alterations which are often associated with elevated intraocular pressure (IOP). Mechanical and vascular causes are implied in the etiology. Mechanistic theory proposes that increased IOP is the most important risk factor in glaucomatous optic neuropathy; however, optic neuropathy can be worsened in patients with well-controlled primary open-angled glaucoma and low IOP [1]. Vascular theory proposes that insufficient ocular blood flow causes optic neuropathy. Vascular dysregulation resulting from vascular endotheliopathy is the most important factor in decreased ocular blood flow [2–4].

Endothelium has a primary role in the regulation of blood flow. In addition to responses to several vasoactive agents and hormones, endothelium itself also releases substances relaxing or constricting vascular smooth muscles such as nitric oxide (NO) or endothelin-1 (ET-1), respectively [5]. Disequilibrium between NO and ET-1 leads to ischemia or vascular dysregulation. It was found that level of ET-1 was increased in the aqueous fluid samples of patients with glaucoma [6, 7].

Several invasive and noninvasive methods are used in the measurement of vascular endothelial function. Endothelium-dependent FMD by occlusion at brachial artery is a noninvasive method which is previously used in the measurement of endothelial function in patients with risk factors (DM, hypertension, hypercholesterolemia, homocysteinemia, etc.) for coronary artery disease. It has been thought that FMD is formed via NO produced by endothelial cells [8]. The aim of the present study using vascular endothelial function measurement to assess FMD was to evaluate relationship between cases with primary open-angled glaucoma and FMD.

## 2. Materials and Methods

The study included 20 patients with POAG and 30 age- and sex-matched healthy controls. The diagnostic criteria for PAOG included IOP >22 mmHg without treatment, open angle at gonioscopy, glaucomatous optic disc changes (cup/disc >0.7 and thinning or pitting at rim), and characteristic visual field defects corresponding to optic disc. The cases with poor SITA reliability indices (false negative errors, false positive errors, and fixation loss exceeding 20%) were excluded. Also, patients with previous ocular trauma or surgery, those with history of steroid use, or those having an eye disorder other than glaucoma were excluded. In addition, patients with systemic diseases such as hypertension, congestive cardiac failure, hypercholesterolemia, diabetes mellitus, cerebrovascular event, or autoimmune diseases were excluded. Control group included healthy individuals without history of medication who presented for routine ophthalmological visits. No perimeter evaluation was performed as they had healthy eyes. All subjects were referred to cardiology clinic for vascular sonography. The study was approved by the local ethics committee and was conducted in accordance with the Declaration of Helsinki, Good Clinical Practice Guidelines. The participants were informed of the nature of the study, and informed consent was obtained from each patient.

In all subjects, blood samples were drawn for measurements of fasting blood glucose, lipid profile, sedimentation rate, and C-reactive protein (CRP). FMD measurements in brachial artery were performed by a cardiologist blinded to clinical characteristics of the patients with 2-dimensional high-resolution ultrasound device (7.0–13.0 MHz, Siemens Medical Sol., Mountain View, CA), as previously described. Imaging study was performed in a silent and dark room at 22–25°C. Ten minutes resting at supine position was provided before measurements and ECG monitoring was applied throughout measurements. Radial artery measurement was performed at a point 3–5 cm superior to antecubital fossa. Transition zone was set at a depth between area at 3 cm depth and closer wall. By magnifying image, intermediate zone between tunica media and adventitia was marked. On pulse Doppler sonography, blood flow velocity and volume were assessed through two-dimensional images generated by signal obtained from center of artery with a sampling angle of 65–70° and sampling distance of 1 mm. A 5-minute occlusion was obtained by inflating cuff up to 250 mmHg at the level of brachial artery. Two-dimensional images of brachial artery were recorded 60 seconds after deflation. Radial artery measurements were recorded during R wave on ECG (end-diastole). Response of brachial artery diameter to hyperemia was calculated as percent increase by using the following formula: 100x [postocclusion diameter (mm) − baseline diameter (mm)/baseline diameter].

Statistical analysis was performed by using SPSS for Windows version 17.0 (Statistical Package for Social Science, Chicago, IL, USA). Normality was tested by using Kolmogorov-Smirnov test. Student's *t*-test was used to assess differences in mean values between groups. *P* < 0.05 was considered significant.

## 3. Results

Table 1 summarizes demographic characteristics and blood measurements of POAG and control groups. There were no significant differences between the groups regarding age, sex, and biochemical measurements.  Table 2 presents vascular parameters in both groups. Percent of FMD increases was 11.9 ± 4.2% and 12.3 ± 4.4% in POAG and control groups, respectively (*P* = 0.86). No significant difference was observed between groups regarding lipid profile and other blood parameters.

## 4. Discussion

Glaucoma represents a group of eye disorders in which many risk factors play a role in its development and progression. IOP is the best known risk factor and reducing IOP is the only available method in the treatment of glaucoma. However, glaucomatous visual field defects can progress despite well-controlled IOP. Thus, it is needed to investigate ocular dynamic in patients with glaucoma to further improve our understanding of ocular dynamics.

Previous studies have reported the role of vascular factors in the development and progression of glaucomatous optic neuropathy in PAOG [9, 10]. These previous works focused on ocular blood blow.

In the present study, it was revealed that there is no impairment in systemic vascular endothelial function in patients with POAG when compared to controls by using brachial artery FMD measurements. Currently, FMD is the gold standard used in the measurement of arterial endothelial function in clinical trials [11]. FMD is high-frequency sonographic visualization of brachial artery that shows endothelial-dependent flow-mediated vasodilation. Hyperemia-induced transient ischemia leads to increased stress in brachial artery wall and it causes vasodilation by promoting endothelial NO release. Noninvasiveness of this technique allows reproducibility of measurements and improved patient adherence. It should be considered that there can be interobserver and intraobserver differences in measurements. However, previous studies showed that inter- and intraobserved differences are minimal without significant effect on results [8, 12]. In our study, measurements were performed by an experienced cardiologist.

FMD measurement is considered an important marker in establishing prognosis and treatment response in coronary artery disease [13]. Endothelium has a major role in the regulation of blood flow. NO and ET-1 are two important vascular regulators which provide vascular tone and activity. Disequilibrium between NO and ET-1 leads to ischemia and vascular dysregulation [7]. At ocular level, ET-1 causes decreased retinal, choroidal, and optic nerve head blood flow and vasoconstriction at posterior ciliary artery. Increased ET-1 levels can play a role in the astrocyte proliferation seen in glaucomatous optic neuropathy [14]. Increasing ET-1 levels in the aqueous humor of patients with POAG reduces outflow of aqueous fluid by causing contraction in trabecular network [15].

In studies on normotensive glaucoma (NTG), it was found that ET-1 levels were increased in both serum and aqueous humor, suggesting both ocular and systemic vascular dysfunction in NTG [16–18]. In studies on patients with PAOG, ET-1 levels were found to be increased in aqueous humor, while no change was observed in serum ET-1 levels [7, 16, 19–22]. This suggests that the role of ET-1 in vascular dysfunction in POAG is localized to tissue. Only Cellini et al. [23] reported increased serum ET-1 levels in selected cases with disease progression despite well-controlled IOP.

In the previous 3 studies using FMD, it was reported that FMD was decreased in patients with POAG [1, 23, 24]. However, there are inconsistencies among these studies. In the study by Su et al., in which patients with NTG and POAG were compared, it was reported that mean percent of FMD increase in patients with NTG was lower than that in patients with POAG [1]. However, Fadini et al. [24] found no significant difference between patients with PAOG and NTG; even authors reported lower percent of FMD increase in POAG group when compared to NTG group. In the study by Cellini et al. [23] patient group consisted of those with progressive visual field defect despite well-controlled IOP; thus, they emphasized vascular theory.

In our study, it was demonstrated that there is no impairment in systemic vascular function of cases with POAG. We think that, in contrast to NTG with systemic component, POAG is a disease specific to the eye. Valuable information could be obtained about systemic vascular function in patients with POAG by designing studies with larger sample size which demonstrate indirect vascular endothelial function such as ET-1 level and number of endothelial progenitor cells.

## Figures and Tables

**Table 1:** Demographic findings and blood measurements.

	POAG (*n* = 20)	Control (*n* = 30)	*P* value
Age	58.25 ± 13.3	58.68 ± 13.6	0.89
Sex (female/male)	8/12	11/19	0.82
Glucose (mg/dL)	90.23 ± 10.2	91.12 ± 11.3	0.78
Total cholesterol (mg/dL)	203.4 ± 23.9	200.7 ± 42.1	0.73
LDL cholesterol (mg/dL)	115.6 ± 22.2	108.1 ± 17.4	0.18
HDL cholesterol (mg/dL)	46.2 ± 7.4	44.7 ± 9.2	0.43
Triglyceride (mg/dL)	177.7 ± 75.2	182.9 ± 81.3	0.69
Sedimentation rate (mm/s)	12.2 ± 9	9.9 ± 8	0.21
C-reactive protein	5.2 ± 2.4	5.7 ± 5.1	0.76

**Table 2:** Vascular parameters.

	POAG (*n* = 20)	Control (*n* = 30)	*P* value
Baseline diameter (mm)	38.6 ± 5.7	37.2 ± 2.9	0.24
Dilatation diameter (mm)	43.2 ± 6.22	41.9 ± 6.2	0.17
FMD %	11.9 ± 4.2	12.3 ± 4.4	0.86
